# Phytochemicals Targeting Estrogen Receptors: Beneficial Rather Than Adverse Effects?

**DOI:** 10.3390/ijms18071381

**Published:** 2017-06-28

**Authors:** Sylvain Lecomte, Florence Demay, François Ferrière, Farzad Pakdel

**Affiliations:** Institut de Recherche en Santé-Environnement-Travail (IRSET), UMR 1085 Inserm, TREC Team, University of Rennes 1, 35000 Rennes, France; sylvain.lecomte@univ-rennes1.fr (S.L.); florence.demay@univ-rennes1.fr (F.D.); francois.ferriere@univ-rennes1.fr (F.F.)

**Keywords:** estrogen receptor, ligand, xenoestrogens, selective estrogen receptor modulators, transcription, epigenetic regulation, cell signaling, cancer

## Abstract

In mammals, the effects of estrogen are mainly mediated by two different estrogen receptors, ERα and ERβ. These proteins are members of the nuclear receptor family, characterized by distinct structural and functional domains, and participate in the regulation of different biological processes, including cell growth, survival and differentiation. The two estrogen receptor (ER) subtypes are generated from two distinct genes and have partially distinct expression patterns. Their activities are modulated differently by a range of natural and synthetic ligands. Some of these ligands show agonistic or antagonistic effects depending on ER subtype and are described as selective ER modulators (SERMs). Accordingly, a few phytochemicals, called phytoestrogens, which are synthesized from plants and vegetables, show low estrogenic activity or anti-estrogenic activity with potentially anti-proliferative effects that offer nutraceutical or pharmacological advantages. These compounds may be used as hormonal substitutes or as complements in breast cancer treatments. In this review, we discuss and summarize the in vitro and in vivo effects of certain phytoestrogens and their potential roles in the interaction with estrogen receptors.

## 1. Introduction

**Figure 1:**
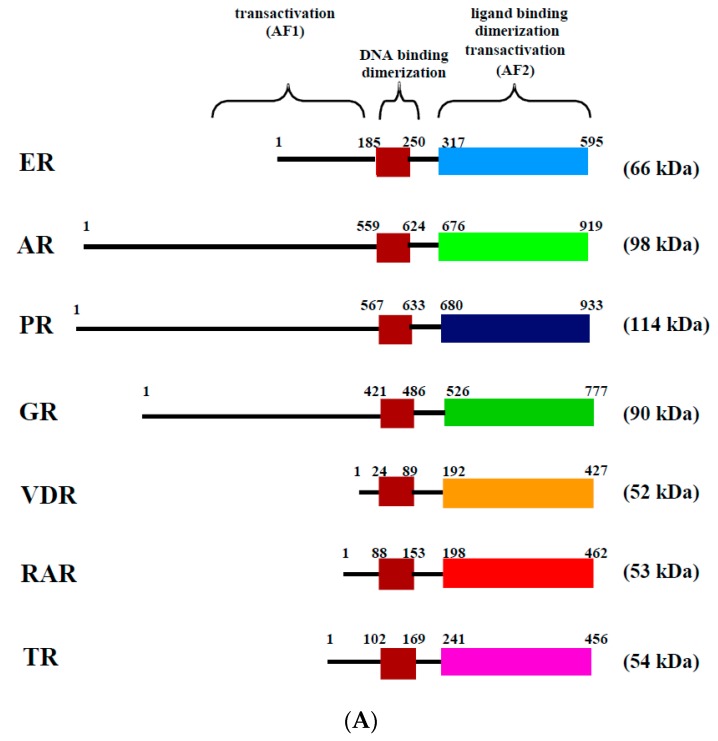

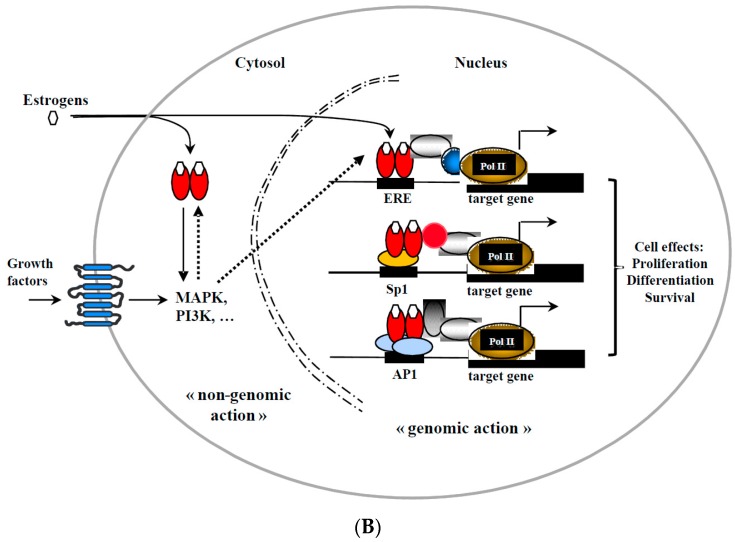
Structure and mechanisms of action of the estrogen receptor (ER). (**A**) The evolutionarily conserved domains of several nuclear receptors, including ER, AR (androgen receptor), PR (progesterone receptor), GR (glucocorticoid receptor), VDR (vitamin D receptor), RAR (retinoid acid receptor) and TR (thyroid receptor). Domains involved in DNA and ligand binding, as well as in dimerization, ligand-independent transactivation function (AF1) and ligand-dependent transactivation function (AF2) are shown. The number of amino acids for each domain is presented. The approximate molecular weight of each nuclear receptor is also indicated on the right side; (**B**) estradiol (E2) mediates multiple phenotypic changes in cells by binding to its receptor. E2 enter the cell through the lipid membranes and binds ER in the cytoplasm or the nucleus. ER mediates E2 effects through diverse transcriptional mechanisms. In the nucleus, the activated ER forms a dimer to tightly fix DNA directly at the ERE sites or indirectly at Sp1 or Ap1 sites. The activated ER is then able to recruit cofactors and RNA polymerase II (pol. II), which allows the transcription of target genes (ER genomic action). Furthermore, ERs can use rapid non-genomic action through the activation of intracellular kinases related or not to the growth factor signaling.

Estrogens, such as 17 β-estradiol (E2), are steroid hormones derived from cholesterol by the successive action of steroidogenic enzymes. They are involved in multiple physiological processes by acting on various tissues. In particular, they participate in the establishment and regulation of the reproductive organs in both males and females, including the gonads or the mammary gland [1]. Furthermore, estrogens participate in many physiological processes in non-reproductive tissues, such as growth and remodeling of bone, differentiation and protection of the central nervous system, vasodilation of cardiovascular systems and lipid metabolism in the liver [1,2]. At the cellular level, E2 has multiple effects, including proliferation, differentiation and survival. E2 is a small, liposoluble molecule that passively enters the cell through the plasma membrane. E2 actions are mainly mediated by their binding to two estrogen receptors, ERα and ERβ, which are localized in the cytoplasm and in the nucleus (Figure 1B). These receptors are members of the nuclear receptor superfamily, which also includes receptors for androgens, progesterone, glucocorticoids, thyroids, retinoid acids, and vitamin D, as well as more than twenty orphan receptors (Figure 1A) [3]. Many nuclear receptors are activated by specific ligands and generally act as transcription factors by binding to specific DNA sequences in the genome. Similar to the other nuclear receptors, ERs are modular proteins that consist of distinct structural and functional domains. The N-terminal domain contains the ligand-independent transactivation function (AF1). The central domain contains the conserved zinc finger DNA-binding-domain, and the C-terminal domain contains the ligand-dependent transactivation function (AF2), as well as the ligand binding and dimerization sequences (Figure 1).

ER-mediated E2 actions at the transcriptional level of the estrogen-sensitive genes are called “genomic” E2 actions. Direct binding of ERs to the chromatin occurs at the estrogen-responsive-element (ERE) at target gene promoters. This induces the mobilization of the transcription coregulators needed to modify chromatin structure and thereby transcriptional regulation of specific gene (Figure 1B). This represents the classical pathway, but many E2-target genes do not contain the ERE. In this case, ERs modulate transcription through DNA-binding sites for Sp1 (stimulating protein 1) or AP1 (activator protein 1) transcription factors [4]. Furthermore, genome-wide studies performed by ChIP (chromatin immunoprecipitation) experiments in breast cancer cell lines specified that ER preferentially regulates its target genes by binding distal regulatory elements [5]. These distal regulatory sites can interact with the promoters of E2 target genes due to chromatin looping. This mechanism of transcriptional regulation represents more than 90% of E2-target genes [5]. Interestingly, these regulatory elements are capable of interacting with several promoters and other enhancers at the same time and are mainly contained in genomic areas called TADs (topologically associating domains) [6,7].

In contrast to the genomic action, the non-genomic actions of estrogens involve cytoplasmic signaling pathways (Figure 1B) and occur rapidly, on the order of seconds or minutes. This leads to the activation of several intracellular signaling pathways such as MAPK (mitogen activated protein kinase) or PI3K (phosphatidylinositide 3-kinase) [8]. Recent studies reported convergence or cross-talk between the genomic and non-genomic actions of ER, enabling a fine regulation of target genes and increasing the complexity of the estrogenic pathways [9,10,11,12].

ERs are generated from two different genes that are localized on chromosome 6, for ERα, and chromosome 14, for ERβ, in humans. The utilization of different promoters and splicing processes results in multiple ER variants that can interfere with the transcriptional activity of wild type ERs in various cell types [13,14,15,16].

Many tissues express both ER subtypes, but with variable expression profiles. For instance, ERα is highly expressed in female reproductive tissues (ovary, womb, mammary gland). ERβ is greatly expressed in ovaries but poorly expressed in the mammary gland. In men, ERα is strongly expressed in the testicle (Leydig cells and gubernaculum), whereas ERβ is found in the prostate, germinal cells and epididymis. On the other hand, both receptors with variable expression levels are found in male and female, lung, hepatic, fat, osseous, and nervous tissues and endothelial cells [1,17,18,19]. Knockout in mice demonstrated crucial roles for both ERα and ERβ during the development of reproductive tissues, gametogenesis, and neuronal growth and differentiation [1,20,21]. The appearance of ER seems to be under spatio-temporal control during development [1,19,20]. For instance, ERα expression has been found in the developing uterus as soon as the 15th day of fetal in mesenchymal cells, while it appears later in the epithelial cells and it rises during the neonatal period. In the cerebral cortex of rodents, ERα expression is greater in postnatal life and decreases substantially during puberty [20]. In the testis under development, ERα is expressed in the gubernaculum, a ligament which differentiates into the cremaster muscle involved in the final positioning of the testis within the scrotum. Its expression is strong between 17 to 20 dpc and barely detectable between 4 and 12 dpp, indicating a role of estrogens and ERα in the right positioning of the testis [19]. However, during mouse brain development, ERβ distribution varies in different areas. ERβ is found mostly in the midbrain and hypothalamus at E12.5, and its expression increases at E15.5 and E16.5. Interestingly, ERβ expression appears intensely and extensively throughout the brain, including in the cerebellum and striatum, at E18.5, whereas very few positive cells may be distinguished in the ventricular region [21].

Many natural and synthetic chemicals in the environment and in food have been reported with hormonal activity, particularly showing estrogenic potency [22]. These compounds are called endocrine disrupting chemicals (EDCs). A lot of EDCs are generated from human activities. For example, polycyclic aromatic hydrocarbons (PAH), such as polychlorinated dibenzo-*p*-dioxins and dibenzofurans, or polychlorinated biphenyls (PCBs), which are the most persistent and widespread in the environment. Bisphenol A, nonylphenol and ethinyl estradiol were also reported to be among the major environmental estrogens. A series of experimental and epidemiological studies over the past decades have suggested that these environmental contaminants can interfere with normal hormonal processes and induce deterioration of the reproduction function in males and females [22,23,24,25,26,27].

Furthermore, numerous natural molecules present in vegetables and plants possess estrogen- and antiestrogen-mimetic activities. These natural molecules are mainly phytoestrogen isoflavones, the most widely consumed. The most abundant isoflavones are genistein and daidzein, which are present in soybean, in particular, and also found in certain fruits, legumes and nuts [28]. Flavones, such as coumestans and lignans are other classes of phytoestrogens, which are also found in certain fruits and legumes [28]. Moreover, some mushrooms, mosses and fungi produce compounds with estrogenic activity. These compounds are called mycoestrogens, such as zearalenone [28]. In this review, we focus on these phytochemicals interacting with ERs and discuss their molecular actions and their potential effects on human health.

## 2. Structure and Sources of the Major Dietary Phytoestrogens

**Figure 2:**
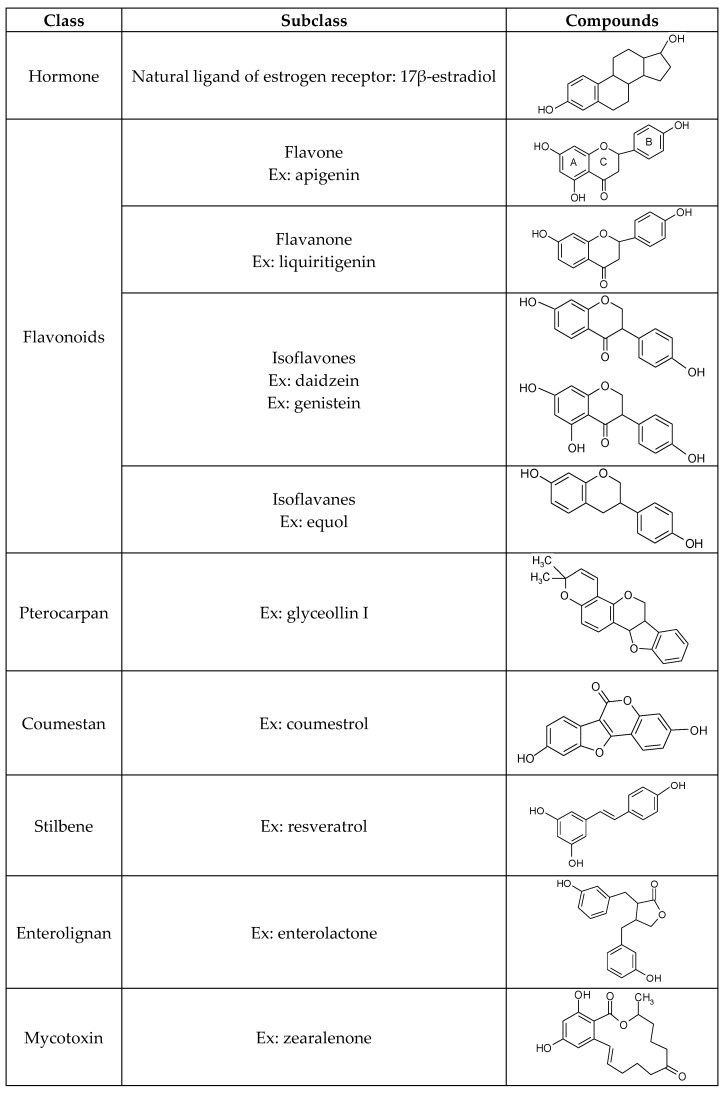
Illustration of the chemical structures of different groups of phytoestrogens. Ex: Example.

Against environmental stresses and aggressions, plants produce secondary metabolites belonging to the large family of polyphenols, which have many biological activities, such as antioxidant, antifungal and antibiotic properties. All of these compounds contain one or several aromatic rings with at least one hydroxyl group. Hydroxyl groups can be free, but most of the time they are engaged in another function with an ester, ether or a glycoside. Among these compounds, phytoestrogens have a structural similarity with 17β-estradiol and could bind both ERs. Phytoestrogens are classified into six groups based on their chemical structures (Figure 2). In this review, we have chosen to present only the aglycone structure of a few phytoestrogens.

### 2.1. Flavonoids

Flavonoids, from *flavus* (yellow in Latin), are pigments of flowers and fruit, and represent the major group. They are formed by 2 aromatic rings bearing at least one hydroxyl group. The aromatic rings, called A and B, are connected by a carbon bridge consisting of three carbons combined with an oxygen to carbons of the A ring. Together, they formed a new 6-ring structure, called C [29] (Figure 2). Flavonoids could be divided into sub-classes depending on the position of the B ring at position 2 for flavones and derivatives and at position 3 for isoflavones and derivatives. Moreover, depending on hydroxylation degree and/or the position of the hydroxyl group, one can distinguish the flavan-3-ols, the flavanones and the flavonols [29].

Here, we have focused on flavones and isoflavones. Flavones are represented by compounds, such as apigenin, found in parsley or chamomile. Apigenin has a beneficial effect on human health [30]. The daily intake of flavones is very low and estimated between 0.3 and 1.6 mg/day [31]. Isoflavones such as genistein or daidzein are found in large quantities in soybean. The daily intake of isoflavones is low in Western countries (0.1–1.2 mg/day) and higher in Asian countries, where they consume more soy product (up to 47 mg/day) [29,32]. Approximately 30% of the population in Western countries and 60% of the population in Asian countries possess gut microbiota able to metabolize daidzein into the isoflavan equol, which shows a greater affinity for ERs than daidzein. Equol exists through two enantiomers, the *R*-(+) equol and the *S*-(−) equol. This latter enantiomer is the natural compound produced by microbiota in human and rat [33].

### 2.2. Pterocarpans

Pterocarpans derive from isoflavones. Their structure is described as a benzo-pyrano-furano-benzene, where the B-rings are coupled to position 4-one [34]. Glyceollins, which correspond to prenylated 6a-hydroxy pterocarpans, are the main delegates of this family [35]. They belong to phytoalexin and are produced from daidzein via an enzymatic pathway, mainly in soybean, by the action of a diversity of elicitors such as UV stress, bacterial or fungi infection [36]. These compounds have been known since the 1970s for their involvement in plant defense [37], but the ability of these compounds to act as phytoestrogens was only established in 2000 [38].

### 2.3. Coumestans

Coumestans are produced by oxidation of pterocarpan [39]. The structure of coumestans consists of a benzoxazole fused to a chromen-2-one [40]. The first, discovered in 1957 by Bickoff et al., and the best documented is coumestrol, which is abundant in alfalfa, soybean and clover [41]. This compound was shown to have a high affinity for both ERs and to induce a response of the same magnitude as that observed with E2 [42].

### 2.4. Stilbenes

Like glyceollins, stilbene belongs to the phytoalexins and participates in plant defense against injury, stress or infection [29]. Resveratrol, the main representative of the stilbene family, is abundant in grape and red wine, with a concentration up to 12 mg/L [43]. Although resveratrol was reported to interact with ERs, its agonist or antagonistic effects remain controversial [44,45].

### 2.5. Lignans

The two best-documented lignans are secoisolariciresinol and matairesinol [46]. Lignans are particularly abundant in flaxseed and sesame seed, and at minor concentrations in cereals, vegetables and fruits. The two major metabolites of lignans in human are also produced by gut microbiota. They present a weak estrogenic action and are called enterolactone and enterodiol [47]. 

### 2.6. Mycoestrogens

Another family of dietary estrogen, called mycoestrogens, is produced by fungi. In this family, the most documented is zearalenone and its derivatives. Zearalenone is produced by *Fusarium* and is found in poorly stored cereals. Zearalenone structure consists of resorcinol moiety fused with a 14-member macrocyclic lactone [48]. According to the European Safety Authority (EFSA), zearalenone is found in 15% of cereals consumed in Europe [49]. Zearalenone has adverse effects on human health, including reprotoxicity [50,51], genotoxicity, and oxidative stress [49]. This chemical and its metabolites, particularly α-zearalenol, which is used as growth promoter in cattle, are able to bind ERs with high affinity and act as strong ERα agonists [51].

## 3. In Vitro Effects of Phytoestrogens

The proliferation of ERα-positive breast cancer cells is enhanced by estrogens, which induce multiple growth factors, cyclins and cytokines involved in cell survival and cell cycle progression. Although ERα has a proliferative effect, ERβ acts as a negative regulator of ERα in breast cancer cells, counteracting the mitogenic effect of estrogens [15,52,53,54]. Interestingly, in many reported ER-selective bioassays, such as the proliferation of breast cancer cell lines, gene reporter assays in mammalian or non-mammalian cells, and ER binding assays, it was found that most phytoestrogens preferentially interact with ERβ and display high specificity toward ERβ transactivation [55,56,57]. Recently, using a fluorescence resonance energy transfer (FRET) assay, Jiang et al. [57] showed that some phytoestrogens, such as genistein, daidzein, equol and liquiritigenin, recruit the coactivator SRC3 much more efficiently to ERβ than to ERα. These data strengthen the ERβ-selectivity of many phytoestrogens. Hence, a relationship between the ERα/ERβ ratio and phytoestrogen effects exists [58,59]. It is suggested that the presence of ERβ is associated with the “good” effect of phytoestrogen whereas a high concentration of phytoestrogen in cells expressing ERα was associated to the “bad” effect of phytoestrogen [60].

Several in vitro studies showed that genistein, the most abundant isoflavone present in soybean, has antiproliferative effects on various cancer cells, including prostate, ovarian, and breast cancer [61,62,63]. While genistein effects can be mediated at least in part by ERβ, other molecular mechanisms, for exemple caspase-3 activation, have been reported to explain growth inhibition or proapoptotic effects of genistein. Additionally, by direct inhibition of tyrosine kinase activities, genistein is also able to prevent cancer cell growth. For example, genistein pretreatment could significantly reduce the activation of Akt kinase by epidermal growth factor (EGF). The inhibition of nuclear factor κB (NF-κB) activity by genistein was also reported in prostate, breast, lung, and pancreatic cancer cells [64,65,66,67,68]. An explanation of this effect is that genistein significantly inhibits Akt kinase activity by decreasing its phosphorylation at serine 473, which can inhibit NF-κB activity [65,69]. Another study reported that the inhibition of prostate cancer cell growth exerted by genistein is linked to a reduction of telomerase activity that is pivotal for cellular proliferation capacity and immortality. [70]. Together, these actions of the isoflavone genistein could contribute to its apoptotic effects in different human cancer cells. It is also interesting to note that in ER-positive MCF-7 cells, the biphasic actions of genistein can be observed with growth stimulation at low concentrations and inhibition at high concentrations. These observations indicate the complexity of the actions of genistein and phytoestrogens globally for their anti-cancer properties.

One of the key mechanisms underlying the maintenance of genome stability and gene expression is DNA methylation. This process occurs on the cytosine of cytosine-guanine dinucleotides (CpG regions). In the human genome, the majority of CpG regions are methylated, except for those located within CpG-rich regions, called CpG islands, which are usually found within gene promoters. Methylation of CpG islands could lead to the inactivation of gene expression by inhibiting the recruitment of transcription factors necessary to induce transcription. Indeed, DNA methylation/demethylation is a dynamic process that allows certain genes to switch ON and OFF at different periods of time. This process appears to be particularly crucial during embryonic development, tumorigenesis, cell division and cell differentiation. For instance, the OCT4 gene, which is essential to maintain pluripotency in embryonic stem cells, becomes methylated in differentiated tissues to avoid unsuitable pluripotency [71]. On the other hand, the loss of expression of tumor suppressor genes by DNA methylation is often observed in cancerous cells. Re-expression of these genes by the inhibition of DNA methyltransferases has provided many successes in the treatment of cancers.

Interestingly, a recent study showed that genistein can reduce DNA methylation in the promoter regions of the Wingless-int (Wnt) genes, which induces the expression of Wnt proteins in colon cancer cells [72]. The Wnt signaling pathway includes a large number of proteins involved in organogenesis and cell-cell adhesion, cell proliferation and differentiation. In addition to its importance in normal cellular physiology, Wnt signaling is also closely involved with carcinogenesis. Notably, the loss of the expression of Wnt proteins by promoter hypermethylation or abnormal activation of Wnt signaling have been detected in the majority of colon tumors and colon cell lines [73,74,75]. It is, therefore, possible that genistein, acting as an inhibitor of the DNA methyltransferase, could be able to induce significant Wnt signaling pathways to protect the development of colon cancer. Another study conducted on the human colon cancer cell lines SW480 and HCT15 showed that genistein blocks cell proliferation in the G2 phase of the cell cycle [76]. The authors showed that the action of genistein-inhibition on cell growth is mediated by overexpression of Dickkopf 1 (DKK1) in SW480 and HCT15 cells treated with genistein. DKK1 is a key regulator of the Wnt signaling pathway that promotes cell differentiation and apoptosis. The repression of the tumor suppressor DKK1 by the hypermethylation of its promoter is reported in various diseases, including colorectal cancer [77,78,79,80]. However, DNA methylation of the DKK1 promoter is not affected by genistein treatment in either cell line. However, genistein induces acetylation of histone H3 within the promoter region of the DKK1 gene in SW480 and HCT15 cells. This indicates that genistein induction of the expression of the DKK1 gene is linked to the increase in histone acetylation [76]. Another recent study showed that genistein is able to epigenetically reactivate ERα in ERα-negative breast cancer models, both in vitro and in vivo [66]. Similarly, in the prostate cancer cell lines LNCaP and LAPC-4, genistein was able to increase the expression of ERβ through decreasing the methylation of the ERβ promoter at physiological ranges (0.5–10 μmol/L) [81]. Hence, genistein could increase the sensitivity of these cancers to endocrine therapies, such as the antiestrogen tamoxifen. In this study, the authors showed that genistein significantly increased histone acetylation patterns in the ERα promoter by inhibiting the enzymatic activities of histone deacetylase (HDAC). It is of interest to note that this effect was enhanced in a synergistic manner when ERα-negative MDA-MB-231 breast cancer cells were co-treated with genistein and TSA, an inhibitor of HDACs. Importantly, the anticancer properties of tamoxifen to inhibit cell growth become much more efficient both in vitro and in vivo, in xenograft nude mice as well as in spontaneous breast tumor mouse models, in the presence of genistein [66]. Together, these studies suggest that in addition to DNA methylation, genistein may also modify histone marks of critical genes to prevent cancer development and progression. These epigenetic actions of genistein mediate the activation of tumor suppressor genes in cancer cell lines but also in animal models. The consequences of endocrine disruptors to different cell types have been widely studied. However, in industrial countries, detectable levels of EDCs were found in human, indicating that people are constantly exposed. Hence, studies on acute effects do not reflect the consequences of constant exposure. Chronic exposure of MCF7 cells with genistein induces a down-regulation of the PI3K/Akt signaling pathway, inhibits the growth-promoting activity of E2 or EGF, and reduces histone H3 acetylation without affecting ER expression. This indicates that chronic treatment leads to epigenetic changes in the cells [82,83].

## 4. In Vivo Effects of Phytoestrogens

The concentration of phytoestrogens in the plasma is considerably different in the human population. For example, in Finnish men the average plasma concentration of genistein is about 0.5 nM whereas it is about 276 nM in Japanese males. However, after absorption of dietary phytoestrogen, a plasmatic peak was detected between 0.2 and 6.5 μM with bioavailability between 5% and 66% [84]. Moreover a pharmacokinetic study performed on postmenopausal women found that the concentration of free genistein could reach 40 nM [85]. Epidemiological studies reveal that a lower risk of breast and prostate cancers is observed in Asians, who consume 20–50 times more soy products than Americans [86]. In vivo experimental studies have also reported that some dietary components, including isoflavones and enterolignans, could inhibit the development of cancers [69,87,88]. This suggests that active molecules in the soybean, such as genistein, daidzein, equol and glycitein, may act as natural chemopreventive agents and could be used against tumor progression in humans. Moreover, clinical studies carried out to assess the effectiveness of isoflavones in patients with prostate cancer found that isoflavone supplementation significantly reduced the expression of the poor prognostic tumoral marker, prostate specific antigen (PSA), and the expression of androgen receptor (AR), but without effecting the expression of ERβ or circulating hormones. These studies have suggested that isoflavones, including genistein and daidzein, may be beneficial in the prevention of prostate cancer by inhibiting the expression of AR and PSA [89,90]. Furthermore, in vivo xenografts in mice model and in vitro studies conducted on androgen-dependent (LNCaP) and androgen-independent (DU145 or PC3) prostate cancer cell lines showed that some phytoestrogens, such as coumestrol, are able to elicit caspase-dependent apoptosis, supporting the hypothesis that phytoestrogens may have anticancer effects in prostate cancer. Conversely, some clinical trials seeking to establish that consumption of phytoestrogens is beneficial in prostate cancer have been inconclusive [91]. For instance, a double-blind trial conducted by Adams and collaborators [92] showed no significant difference in PSA among men who did or did not consume a diet rich in isoflavones for 12 months. Although this study is limited by the relatively small number of patients, it could indicate that the period and duration of treatment may be essential for the anticancer effects of isoflavones. While the exact mechanisms by which isoflavones can prevent the development or progression of prostate cancer remain unclear, many mechanisms have been proposed, including the regulation of genes involved in the cell cycle, such as an upregulation of p21 resulting in cell cycle arrest at the G1/S phase, apoptosis, antioxidant effects, DNA repair, inhibition of angiogenesis and metastasis, and also the antagonism of estrogen and androgen signaling pathways (for review see [93]). It should be noted, therefore, that changes in some signaling pathways or in the expression of key enzymes involved in steroid metabolism during different stages of prostate cancer could play an essential role in the effects of phytoestrogens.

In vivo studies carried out in a true physiological context in humans and animals have indicated that the food content of isoflavones poses no safety issue, as generally consumed in diets based on soy products [94]. Moreover, the concentration of isoflavones varies considerably depending on the place of soybean cultivation (from 85 mg/100 g in Taiwan to 178 mg/100 g in Korea) or according to the culinary process (6 mg/100 g 195 mg/100 g in Foojook soup) [95]. A similar observation was made in lyophilized cabbage compared to fermented cabbage [96]. As the structure of the major isoflavone compounds is close to that of E2 and because these compounds are known to have weak estrogenic activities, the possible effects of some isoflavones on estrogen-target tissues and on reproductive function have been extensively explored [97,98,99,100]. However, there are conflicting results regarding the effects of isoflavones on reproductive function because the long-term studies on the impact of these compounds on the development and function of reproductive tissues are not sufficient. In addition, comparisons between different studies are complicated because there are differences in the experimental design, such as the physiological state of the animal, the presence of circulating hormone, and the duration, doses and methods of exposure (injection or gavage). More importantly, differences in the metabolism of isoflavones between animal models and humans can also give inconclusive results. Thus, all these parameters must be considered when assessing the impact of isoflavones on reproductive function. All major soybean isoflavones, genistein, daidzein, equol and glycitein, were reported to be estrogenic in the mouse or rat uterine growth assay [97,98]. For instance, 100 mg/kg body weight of genistein or equol, administered by gavage for 4 successive days (post-natal at day 17–20), was found to significantly increase uterine weights and the expression of ERα in the uterus [97]. Another study compared the estrogenic potential of several phytoestrogens, including genistein, daidzein and coumestrol in immature mice using different morphological and biochemical tests on the uterus. Interestingly, while certain compounds, such as genistein and coumestrol, showed estrogenic activity in all tests, others showed estrogenicity in only a single test or did not show estrogenicity in any test [101].

The estrogenic potency of isoflavones was also assessed, in vivo, in several non-mammalian model organisms. For example, goldfish or medaka fed for several weeks with a diet containing coumestrol or genistein showed an increased production of the vitellogenin, an egg yolk protein precursor, which is normally produced in the liver under estrogenic stimulation. However, no adverse effects on reproduction function (fecundity and fertility) have been reported [102,103], indicating that the production of vitellogenin may serve as an indicator of estrogen exposure but not as an indicator of reproductive dysfunction by estrogen exposure. In contrast, a recent study from Bennetau-Pellisero and collaborators reported [104] that goldfish fed soybean meal for 20 weeks after hatching show a reduction in fertility success and larvae production. Particularly, both male and female fish groups displayed changes in the plasma testosterone and E2 levels, as well as in their spermatogenesis process and oocyte maturation.

Concerning male reproductive function, a study has been conducted on marmosets fed with soy-based milk during the first six weeks of life and compared to animals fed with a standard cow’s milk-based diet [105]. This study reported that soy-fed marmosets had body weights, organ weights (prostate, seminal vesicles, pituitary, thymus and spleen) and penis lengths comparable to the other animals. Although lower blood testosterone and higher Sertoli and Leydig cell numbers per testis were observed in soy-fed marmosets, no adverse reproductive consequences were detected in adulthood, including the timing of puberty and overall fertility [105]. On the other hand, Adachi et al. have carried out a toxicogenomic analysis in mice to investigate long-term effects of neonatal exposure to genistein on testicular gene expression. In addition, the authors used diethylstilbestrol (DES), known as a potent estrogen, as a positive control because exposure to DES has been reported to induce morphological changes and alteration of gene expression in reproductive organs. Male mice fed with genistein (1000 μg/mouse/day) from days 1–6 after birth did not show any morphological changes in testes at 12 weeks of age, despite decreased ER and AR gene expression. As expected, DES (50 μg/mouse/day) did show gene expression and morphological changes in testes at 12 weeks of age [106]. This suggests that neonatal exposure to genistein has no long-term effects, according to this analysis. 

Following menopause in women, there is more brain-related pathology, incidence of stroke and loss of bone mass observed than in men [107]. Because estrogens are neuroprotective agents that are involved in bone remolding, one possible explanation may be the decline in estrogen levels. Isoflavones are generally considered to have beneficial effects on bone and brain, although controversial results have been published [108]. In ovariectomized rats, genistein showed a weak osteoprotective effect by promoting bone mineral density [109,110]. Similarly, coumestrol showed a neuroprotection effect in ovariectomized rats subjected to global ischemia [111]. Interestingly, using an ER antagonist, the authors showed that the neuroprotective actions of coumestrol are only partially abolished, suggesting that in addition to classical ER signaling, coumestrol may act via other cellular pathways. Thus, the beneficial effects of isoflavones may depend on the quantity and ratio of the expression of ER subtypes, the endogenous steroid hormones and period of life [111]. Moreover, it would be interesting to find out the cellular targets of coumestrol mediating its neuroprotective action. These cellular pathways could be used in the therapeutic potential of coumestrol in the treatment of pathologies related to the central nervous system.

There is growing evidence suggesting that during critical windows of prenatal and postnatal development, environmental chemicals can induce epigenetic modifications, affecting gene expression and consequently impacting developmental pathways. Importantly, it has been suggested that the effects of some environmental chemicals could act across generations, leading to phenotypic and physiological variation in the development and behavior of offspring. The transmission can be a consequence of changes in the transcriptome and epigenome programming within germ cells. While these effects have been recently reported for a number of environmental compounds, such as vinclozolin, atrazine, bisphenol A (BPA), DES, and dioxine [112,113,114,115,116,117,118], studies on the potential transgenerational effects of phytoestrogens are very rare, if any, and need additional work [69,115].

Although the situation is different for phytoestrogens produced by plants, it is worth noting that some fungi also produce compounds, called mycotoxins, with estrogenic properties. For example, zearalenone and fusarin C act as estrogen agonists and are classified as mycoestrogens. These compounds, which could contaminate improperly stored grains, have been linked to increased cancer rates. In vitro, fusarin C, as well as zearalenone and its metabolites, can stimulate the growth and proliferation of human breast tumor cells [119,120,121]. Moreover, in vivo exposure of rats to environmental doses of zearalenone in the last two to three weeks of fetal development and the first days after birth resulted in long-term changes in the development of the mammary gland associated with increased risk for the development of mammary tumors [122].

## 5. Conclusions

**Figure 3:**
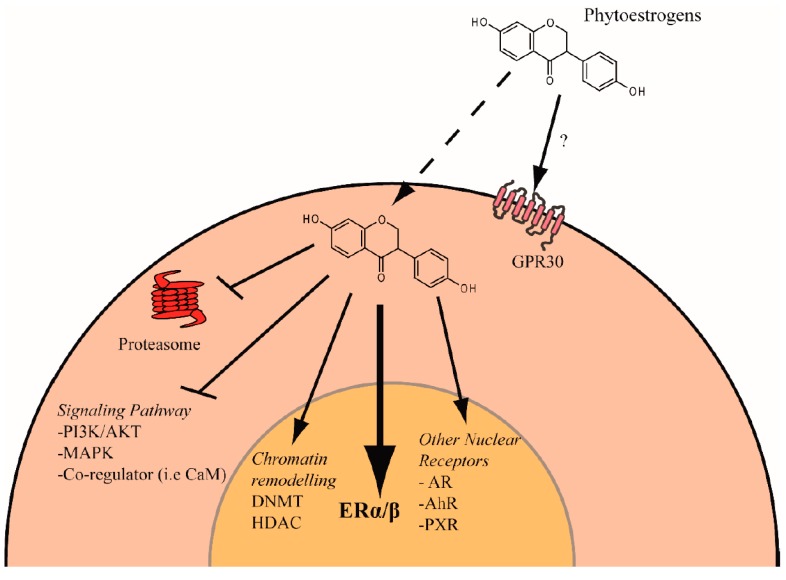
Different targets of phytoestrogens in cells. Cell signaling pathways for estrogens through the nuclear receptors ERα, ERβ and the transmembrane receptor G-protein-coupled ER (GPER; formerly known as GPR30) [136] are shown. Phytoestrogens are able to inhibit mitogenic pathways via ERα or PI3K/MAPK, which in turn inhibit cancer cell proliferation and invasion by modifying AKT, p70S6K and ERK1/2 phosphorylation as well as interaction between ERα with various coregulatory proteins such as calmodulin (CaM). Activation of ERβ inhibits dedifferentiation pathways and induces apoptosis and cell cycle arrest. GPER activation is anti-tumorigenic, as it upregulates p21 and induces cell cycle arrest in prostate cancer [137]. Epigenetic modifications by phytoestrogens, such as demethylation of CpG islands within the promoters of tumor suppressor genes, could contribute to cell growth arrest. Inhibition of proteasomes by phytoestrogens also appears to be another mechanism of phytoestrogen activity in decreasing cancer cell survival.

Although more research is needed, it is clear that some natural compounds from plants, such as phytoestrogens, could have beneficial effects on certain diseases, such as cancer or neurodegenerative diseases. However, in vitro or in vivo studies to analyze the final effects of phytoestrogens may be quite different at low (<1 μM) or high concentrations (>10 μM). For instance, at low doses (from 10 nM to 1 μM), genistein showed mitogenic effects on breast cancer cell growth, whereas at higher concentrations (>10 μM), it showed antiproliferative effects [123,124]. Some of these effects are explained by their interactions with ER subtypes. The ratios and the expressions of ERα and ERβ are different in various tissues depending on the period of life. ERα is mostly expressed in tissues such as the mammary gland, uterus, liver and pituitary, while ERβ is expressed in tissues such as the brain, bone and bladder. Moreover, the abilities of ER subtypes to recruit cofactors, regulate gene expression and stimulate or inhibit cell growth are slightly different. Therefore, in vivo, phytoestrogens may have a complex role, acting as weak estrogens and antiestrogens depending on the tissue. Furthermore, it is believed that the signaling pathways induced by phytoestrogens are not completely identical to those induced by estrogens. As illustrated in Figure 3, phytoestrogens may have different mechanisms of action; therefore, some of these compounds could be considered therapeutic agents and used alone or in combination with usual hormone therapies. For example, the protective effect of isoflavones on prostate cancer may be related to their effects on metabolic pathways involved in androgen and estrogen synthesis [125] or to their epigenetic modifications of DNA, such as the demethylation of CpG islands within the promoters of tumor suppressor genes [81,126,127]. On the other hand, the phytoestrogen coumestrol, which exhibits an important cancer-preventive effect in estrogen-responsive carcinomas, was recently reported to inhibit epithelial ovarian cancer proliferation and invasion by modifying AKT, p70S6K and ERK1/2 phosphorylation [128]. Moreover, previous studies showed an antagonistic effect of genistein and apigenin against the association of ERα with the ubiquitous calcium-dependent protein, calmodulin (CaM). By interacting with ERα, CaM plays a key role in the stabilization and transcriptional activity of ERα dimers at the ERE. The agonistic effect of genistein and apigenin in this interaction may also account for the anti-tumor origin of these compounds against ER-positive breast cancers [129,130]. It is, therefore, essential to continue advances in the understanding diverse signaling pathways activated by phytoestrogens, to fully exploit their anticancer properties and/or their potential roles in estrogen-related diseases. Accordingly, it should also be remembered that changes in the expression or activity of nuclear and membrane receptors for steroids and growth factors, as well as key steroid synthesis enzymes, during cancer progression could play crucial roles in the effects of phytoestrogens (Figure 3). Indeed, flavonoids, especially flavones (ex: luteolin) and flavanones (ex: naringenin), are described as potent inhibitors of aromatase activity [131]. Aromatase is the main enzyme that participates in the transformation of testosterone into estradiol and is hence involved in breast cancer pathology. Moreover, luteolin was also shown to downregulate aromatase gene expression [131]. Phytoestrogens are also able to inhibit proteasome [132], which appears to be essential for breast cancer cell survival [133]. For example, apigenin is capable of inhibiting the catalytic activity of proteasomes, leading to stabilization of ERβ and apoptosis of prostate cancer cells [134].

An important application of phytoestrogens is that they could be used as an alternative to the synthetic selective estrogen receptor modulators (SERMs), which exhibit estrogen agonist or antagonist activity in a tissue-specific manner. Indeed, SERMs are used in the treatment of some estrogen-associated pathologies, such as breast cancer, brain diseases, osteoporosis and menopausal symptoms. In other words, the challenge is to minimize the adverse effects of ER (mitogenic effect) without reducing the beneficial effects (protective effects), such as the control of cell differentiation, neuroprotection, anti-osteoporosis effects, and anti-oxidant activity. Our recent study screening the SERM activity of these compounds revealed a beneficial effect of apigenin and resveratrol, whereas zearalenone has been characterized as having a strong ER-agonist property in breast cancer cell lines and having adverse effects in neuritogenesis [135].

Although recent studies have reported that certain environmental agents caused epigenetic effects that could act across generations, leading to physiological changes of the offspring, there are no examples of perinatal exposure to phytoestrogens at environmentally relevant doses. There is still a need to understand the molecular mechanisms and to investigate how these compounds can influence epigenetic patterns during development.

In this review, we have discussed the effects of phytoestrogens used alone. However, populations are exposed to several compounds at the same time. Thus, it might be important to perform studies of the effect of mixtures of botanical estrogen on human health to improve recommendations for public health.
